# Relation of insulin resistance with social-demographics, adiposity and behavioral factors in non-diabetic adult Canadians

**DOI:** 10.1186/s40200-016-0253-7

**Published:** 2016-08-11

**Authors:** Sai Yi Pan, Margaret de Groh, Alfred Aziz, Howard Morrison

**Affiliations:** 1 Science Integration and Social Determinant Directorate, Public Health Agency of Canada, 785 Carling Avenue, AL 6809B, Ottawa, ON K1A 0K9 Canada; 2 Food Directorate, Health Products and Food Branch, Health Canada, Ottawa, ON Canada

**Keywords:** Insulin resistance, HOMA-IR index, Waist circumference, Waist-to-height ratio, Waist-to-hip ratio, BMI

## Abstract

**Background:**

Insulin resistance is a pathogenic factor for type II diabetes and has been associated with metabolic abnormalities and adverse clinical outcomes. The purpose of this study was to examine the relationship between insulin resistance and socio-demographics, adiposity and behavioral factors in the general, non-diabetic adult Canadian population.

**Methods:**

Data for 3515 non-diabetic adults aged 18 to 79 years from the Canadian Health Measures Survey (cycles 1 and 2, 2007–2011) were analyzed. Insulin resistance index was measured by the homeostasis model assessment of insulin resistance (HOMA-IR), and insulin resistance (IR) was defined as individuals in the highest quartile of the HOMA-IR index. Logistic regression models were used to examine the effect of demographics, lifestyle factors and adiposity measurements on HOMA-IR.

**Results:**

The risk of IR increased with age, particularly in men. Individuals had adjusted odds ratio (OR) (with corresponding 95 % confidence interval) of 5.97 (2.90–8.52) and 25.12 (15.20–41.51) associated with a body-mass-index (BMI) between 25.0 and < 30.0, or ≥30.0, of 9.23 (6.52–13.07) with abdominal obesity (waist circumstance ≥102 cm for men and ≥ 88 cm for women), of 8.72 (6.13–12.39) with a high waist-to-height ratio (>0.57), and of 6.30 (4.33–9.16) with a high waist-to-hip ratio (>0.90 for men and >0.85 for women). Physically inactive people and non-alcohol consumer also had a significantly higher odd of IR.

**Conclusions:**

This study found that men and older, obese and physically inactive people were at increased risk for IR. Adiposity indices including BMI, waist circumstance, waist-to-height ratio and waist-to-hip ratio were highly associated with IR with similar magnitude of association.

## Background

Insulin resistance (IR) is defined as decreased sensitivity or responsiveness to the metabolic actions of insulin, such as insulin-mediated glucose disposal and inhibition of hepatic glucose production [1]. Evidence has accumulated showing that insulin resistance is a pathogenic factor for type II diabetes [2–6], with which about 2.5 million Canadians have been diagnosed in 2010, with an estimated economic burden of $12.2 billion including $2.1 billion of direct cost and $10.1 billion of indirect cost) in 2010 [7]. It has also been associated with increased risk of a number of metabolic abnormalities and adverse clinical outcomes, such as essential hypertension, atherogenesis, coronary heart disease, stroke, and systemic inflammation [2, 8–13].

It has been suggested that IR and subsequent compensatory hyperinsulinemia develops earlier than β-cell dysfunction because insulin secretion in insulin-resistant, non-diabetic persons is increased in proportion to the severity of the insulin resistance even though glucose tolerance remains normal. Therefore, IR might exist and progress before diabetes, and pre-diabetes would be detected by impaired fasting glucose or impaired glucose tolerance [6]. Thus, early identification of individuals with IR may be a way to guide earlier intervention strategies (i.e., prior to the emergence of impaired glucose tolerance) to prevent or delay diabetes onset and related chronic diseases.

The gold standard for assessing insulin resistance has been euglycemic-hyperinsulinemic glucose clamp [14, 15]. This method is invasive, complex and expensive; therefore, it has been of limited use in epidemiological studies. Instead, the Homeostasis Model Assessment of Insulin Resistance (HOMA-IR) is a simpler and more practical method to measure insulin resistance and has been widely used in large epidemiological studies [16]. The HOMA-IR index has been validated as an acceptable proxy measure of insulin resistance in both normal and diabetic people with a good correlation (correlation coefficient: 0.73–0.88) between estimates of IR derived from HOMA and from the euglycemic clamp, and with a good correlation (correlation coefficient: 0.62–0.90) between estimates of β-cell function using HOMA and estimates using continuous infusion glucose model assessment, hyperinsulinemic clamps, the acute insulin response from the intravenous glucose tolerance test [16–18].

There are no report on HOMA-IR and its association with lifestyle factors in the Canadian population. Therefore, the main purpose of this study was to assess the association of IR with demographic and lifestyle factors using a sample of non-diabetic adult Canadians.

## Methods

### Data source and study population

This analysis was based on data from the Canadian Health Measures Survey (CHMS), cycle 1 (2007–2009) and cycle 2 (2009–2011), which was collected by Statistics Canada. The CHMS is an ongoing comprehensive, direct health measures survey, developed to address important data gaps and limitations in existing health information. It provides national estimates at the time of the survey. Ethnics approval was obtained from Health Canada’s Research Ethics Board [19]. Cycle 1 covers approximately 96.3 % of the Canadian population aged 6 to 79 living at home and residing in the 10 provinces and 3 territories. Cycle 2 covered the population aged 3 to 79 living at home and residing in the 10 provinces and 3 territories, and represented more than 96 % of the population. Excluded from all cycles of the CHMS are individuals living on reserves or in certain remote areas, institutional residents, and full-time members of the Canadian Forces. Study design, study subjects, and data collection methods have been described elsewhere [20–23]. The overall response rates were 52 % for cycle 1 and 55.5 % for cycle 2 after adjusting for the sampling strategy, and reflecting the proportion of A) households that agreed to participate (70 % for cycle 1 and 75.9 % for cycle 2); B) selected household respondents that participated in the survey (88 % for cycle 1 and 90.5 % for cycle 2); and C) participants who reported to the mobile examination centre (85 % for cycle 1 and 81.7 % for cycle 2).

Measures for fasting insulin and fasting glucose were available for 3734 (1805 for cycle 1 and 1929 for cycle 2) adults aged 18 to 79 years old. Individuals who were diagnosed by a physician as having diabetes or who had fasting glucose levels above 6.9 mmol/L (the level for operationally defining type II diabetes) were excluded from the analysis (*n* = 219). Therefore, results are based on a sample of 3515 (1716 for cycle 1 and 1799 for cycle 2) adults age 18 to 79 without diabetes.

#### Data collection procedure

During an initial household survey, the CHMS collects self-reported information on socio-demographics, medical history (including current medication use), current health status, and lifestyle behaviours. On an appointed date after the household interview, physical measurements, such as height, weight, waist circumference (WC), blood pressure, and heart rate, were obtained at a mobile examination centre. A sample of blood and urine was also collected from participants for further tests, with approximately one-third of the participants asked to fast for at least 10 h before their clinic visit. A wide range of biomeasures were assessed, such as hemoglobin A1c (HbA1c), high-density lipoprotein (HDL), vitamin D, etc. Fasting participants had additional blood measures available, including triglycerides (TG), insulin, glucose, apolipoprotein A, apolipoprotein B, and low density lipoprotein.

### Key measures

#### Outcome - Insulin resistance (IR)

IR was based on the homeostatic model assessment (HOMA) [16]. HOMA index was determined by the fasting insulin concentration and fasting glucose concentration and defined as:

$$ \mathrm{HOMA}\ \mathrm{index} = \left[\mathrm{fasting}\ \mathrm{insulin}\ \left(\upmu \mathrm{U}/\mathrm{ml}\right)\ *\ \mathrm{fasting}\ \mathrm{glucose}\ \left(\mathrm{mmol}/\mathrm{L}\right)\right]/22.5. $$

Insulin concentration was measured by solid-phase, two-site chemiluminescent immunometric assay. Fasting glucose concentration was measured by the VITROS GLU Slide method (colorimetric). Because there are no universally established cut-offs for classifying IR, as a normal practice, individuals in the highest quartile on the HOMA index (i.e., 25 % of the population) were classified as IR, with the remaining 75 % of the population classified as non-IR [24].

#### Adiposity measures

##### Body mass index (BMI) (kg/m^2^)

BMI was calculated from measured weight and height. Based on BMI, subjects were classified as underweight (<18.5), normal weight (18.5– < 25.0), overweight (25.0– < 30.0), obese (≥30.0) [25].

##### Waist circumference (WC)

A WC ≥102 cm in men and ≥ 88 cm in women was used to identify those with excess adiposity, i.e. abdominal obesity [25].

##### Waist-to-height ratio (WHtR)

WHtR was calculated by dividing waist circumference in centimeter by height in centimeters. A WHtR under 0.570 is generally considered healthy and a WHtR of 0.570 and over (is considered to be risk equivalent to BMI of 30) was used to identify those of excess adiposity [26].

##### Waist-to-hip ratio (WHR)

WHR was calculated by dividing waist circumference in centimeter by hip circumference in centimeters. A WHR >0.90 for men and >0.85 for women was used to identify those with excess adiposity [25].

#### Socio-demographics

##### Education

Individuals were classified as 4 categories according to their highest level of education: less than secondary school graduation, secondary school graduation without post-secondary education, some post-secondary and post-secondary graduation (including trade certificate, or diploma from a vocational school or apprenticeship training, non-university certificate or diploma from a community college, university certificate below bachelor’s level, bachelor’s degree, university degree or certificate above bachelor’s degree). The 4 categories were defined by Statistics Canada [22, 23].

##### Family income adequacy

Individuals were classified as 4 groups based on total household income (Canadian dollars) and the number of people living in the household, which was defined by Statistics Canada: lowest income group, lower middle income group, upper middle income group and highest income group [22, 23]. The definition is as below:

**Table:** 

	# of people in the household	Total household income
Lowest income group	1, 2	<$15,000
	3, 4	<$20,000
	>4	<$30,000
Lower middle group	1, 2	$15,000– < $30,000
	3, 4	$20,000– < $40,000
	>4	$30,000– < $60,000
Upper middle group	1, 2	$30,000– < $60,000
	3, 4	$40,000– < $80,000
	>4	$60,000– < $80,000
Highest group	1, 2	≥$60,000
	>2	≥$80,000

#### Behavioural factors

##### Physical activity index

It was based on total daily energy expenditure values calculated from self-reported responses to questions about the frequency and duration of leisure-time physical activity in the past 3 months [22]. These activities include walking, garden/yard work, swimming, bicycling, dance, home exercises, ice hockey, ice skating, rollerblading, jogging/running, golfing, aerobics, ski/snowboard, bowling, baseball/softball, tennis, weight training, fishing, volleyball, basketball, soccer and any other activities. Individuals were classified as being “active”, “moderate” or “inactive” based on total daily energy expenditure values (kcal/kg/day): > = 3, 1.5– < 3 or 0– < 1.5.

##### Alcohol consumption (daily drinks)

Individuals who did not have at least one drink for the last 12 months were classified as non-drinker, and those who had an average of one drink daily as light drinkers, while those who had an average of two drinks daily were classified as moderate drinkers and 3 or more as heavy drinkers.

##### Smoking status

Individuals who have never smoked were classified as never smoker, and those who were former daily smokers and former occasional smokers as former smokers, while those who were daily smokers and occasional smokers were classed as current smokers.

### Statistical analysis

Data were analyzed using SAS Enterprise Guide version 4 (Cary, NC). HOMA-IR index level was estimated in the population by gender and other demographic factors, and weighted to reflect the Canadian population aged 18 to 79 years (using a bootstrap procedure, with 24° of freedom [22, 27]). Associations of socio-demographics, some behavioural factors (physical activity, alcohol consumption and smoking) and adiposity measures such as BMI, abdominal obesity, waist-to-height ratio, waist-to-hip ratio, with IR prevalence were also examined using odds ratios by logistic regression model. Variables included in the regression models as potential confounders were age (continuous), sex, education (less than secondary, secondary graduate and other post-secondary and post-secondary graduate), BMI (continuous), physical activity index (active, moderately active and inactive), alcohol consumption (non, light, moderate and heavy drinkers) and smoking status (never, former and current). The variable being assessed was adjusted to all above variables except for the variable under consideration. For example, when physical activity was assessed, variables included in the regression models as confounders were age, sex, education, BMI, alcohol consumption and smoking status. However, BMI, WC, WHtR, WHR were not adjusted for each other because their high correlation.

In order to determine the strongest relationship with IR among the four measures of obesity, we standardized the four continuous variables (BMI, WC, WHtR and WHR), such that we could compare the ORs based one standard deviation change. This standardization was done by the STANDARD PROC of SAS software and the standardized continuous variables were entered into the logistic regression models.

In addition, we also performed the analyses by sex to examine whether there is a difference between women and men on the association of various factors with IR.

## Results

Table 1 shows the characteristics of the study population. Of the 3515 subjects included in the analysis, there were 1646 men and 1869 women. Men and women were similar in mean age, BMI, waist-to-height ratio and waist-to-hip ratio. However, men were less likely to be defined as abdominally obese (based on WC) but more likely to have a high waist-to-hip ratio and to be overweight (as defined by BMI), compared to women. Obesity rates, based on BMI were similar for men and women (22.03 vs 21.84 %, respectively). More men than women were in the highest household income adequacy category (52.11 vs 44.88 %) and were heavy drinkers (12.96 vs 1.79 %).

**Table 1:** Characteristics of the study population (≥18 years old), Canadian Health Measures Survey, Cycle 1 & 2, 2007–2011

Variable	All		Men		Women	
	N	%	N	%	N	%
Age (years) (mean (SD))		45.92 (16.70)		46.12 (17.02)		45.74 (16.41)
18–35 years (%)	1058	33.75	488	34.78	570	32.76
36–50 years (%)	1108	32.37	515	32.42	593	32.32
51–79 years (%)	1349	33.88	643	32.79	706	34.92
BMI (kg/m^2^) (mean (SD))		27.48 (7.69)		27.49 (5.55)		27.48 (9.18)
Waist circumference (cm) (mean (SD))		90.87 (15.42)		95.63 (14.39)		86.65 (15.08)
Waist-to-height ratio (cm) (mean (SD))		0.54 (0.09)		0.54 (0.08)		0.53 (0.09)
Waist -to-hip ratio (mean (SD))		0.95 (0.77)		0.95 (0.39)		0.94 (0.99)
Fasting glucose (mmol/L) (mean (SD))		5.03 (0.75)		5.18 (0.79)		4.89 (0.68)
Fasting insulin (μIU/ml) (mean (SD))		9.24 (7.00)		9.77 (7.25)		8.77 (6.74)
HOMA-IR index (mean (SD))		2.15 (1.90)		2.34 (2.06)		1.98 (1.72)
Education level						
Less than secondary	437	12.19	222	13.82	215	10.62
Secondary graduate	556	16.15	243	14.96	313	17.28
Other post-secondary	357	9.77	178	9.79	179	9.74
Post-secondary graduate	2133	60.42	984	59.27	1149	61.53
Not stated	32	1.48	19	2.16	13	0.82
Household income adequacy						
Lowest	181	3.65	65	2.73	116	4.54
Lower middle	512	15.09	215	15.14	297	15.04
Upper middle	1089	29.83	485	27.62	604	31.95
Highest	1618	48.42	833	52.11	785	44.88
Not stated	115	3.01	48	2.41	67	3.59
BMI (kg/m2)						
< 18.5	57	2.11	17	1.92	40	2.3
18.5– < 25.0	1316	39.8	511	32.2	805	47.16
25.0– < 30.0	1288	36.15	726	43.85	562	28.7
≥ 30.0	835	21.93	390	22.03	445	21.84
Waist circumference (cm)						
< 102 in men or < 88 in women	2261	67.77	1161	73.84	1100	61.97
≥ 102 in men or ≥ 88 in women	1245	32.23	481	26.16	764	38.03
Waist-height ratio						
≤ 0.570	2336	70.37	1070	69.07	1266	71.64
> 0.570	1148	29.63	570	30.93	578	28.36
Waist-hip ratio						
≤ 0.90 in men or ≤0.85 or women	1726	50.56	597	39.23	1129	61.41
> 0.90 in men or >0.85 in women	1764	49.44	1046	60.77	718	38.59
Alcohol consumption (daily drinks)						
None	1666	55.26	652	46.29	1014	64.57
Light (1 drink/day)	816	28.09	419	29.03	397	27.11
Moderate (2 drinks/day)	283	9.17	190	11.72	93	6.53
Heavy (≥3 drinks/day)	207	7.48	178	12.96	29	1.79
Smoking status						
Never	1778	50.86	784	48.08	994	53.51
Former	1034	27.34	511	29.37	523	25.4
Current	695	21.8	344	22.55	351	21.09
Physical activity index						
Active	800	21.18	438	24.4	362	18.09
Moderate	909	25.62	448	26.07	461	25.19
Inactive	1806	53.2	760	49.53	1046	56.72

*HOMA* homeostasis model assessment, *BMI* body mass index

Table 2 displays the unadjusted and adjusted odds ratios (OR) of IR associated with demographic and lifestyle factors as well as adiposity measures. People older than 50 years had a significantly higher OR for IR in comparison with younger people. Also, men had a higher OR for IR than women. Compared with normal weight individuals, overweight (BMI: 25.0– < 30.0) and obese (BMI ≥ 30.0) adults had adjusted ORs (95 % CI) of 5.97 (2.90–8.52) and 25.12 (15.20–41.50), respectively. Abdominal obesity (based on WC) was also associated with an increased OR of 9.23 (95 % CI: 6.52–13.07) for IR. Similarly, persons with high waist-to-height ratio (WHtR > 0.570) and waist-to-hip ratio (WHR > 0.90 for men and >0.85 for women) were at increased OR for IR (8.72 and 6.30, respectively). However, these measures of obesity were not adjusted for each other. In addition, compared with physically active people, those who were physically inactive and moderately active were both associated with increased risk for IR (OR = 2.44, 95 % CI: 1.61–3.68 and OR = 2.29, 95 % CI: 1.47–3.55). For alcohol consumption, individuals who were light, moderate and heavy drinkers had decreased ORs for IR compared to those who never drank alcohol. Nevertheless, there were no statistically significant differences for ORs associated with IR for education level, family income adequacy, and smoking status, although there were tendencies of decreasing OR for IR with increasing education level and family income adequacy.

**Table 2:** Odds ratios of insulin resistance associated with demographics, adiposity and behavioral factors, CHMS, Cycle 1 & 2, 2007–2011

Variables	Unadjusted		Adjusted **	
	OR (95 % CI)	*P* for trend	OR (95 % CI)	*P* for trend
Age		0.0001		0.0001
18–35	ref		ref	
36–50	1.05 (0.70–1.57)		0.85 (0.54–1.33)	
51–79	2.16 (1.53–3.03)		1.91 (1.41–2.58)	
Sex				
Female	ref		ref	
Male	1.30 (0.96–1.75)		1.43 (1.08–1.90)	
Education				0.1026
Less than secondary	ref	0.0699	ref	
Secondary graduate	0.80 (0.50–1.29)		0.86 (0.52–1.41)	
Other post-secondary	0.48 (0.28–0.82)		0.79 (0.44–1.43)	
Post-secondary graduate	0.52 (0.33–0.83)		0.66 (0.42–1.04)	
Family income adequacy		0.1305		0.0773
Lowest	ref		ref	
Lower middle	1.57 (0.92–2.68)		1.15 (0.47–2.35)	
Upper middle	0.84 (0.60–1.18)		0.61 (0.39–0.96)	
Highest	0.92 (0.57–1.49)		0.67 (0.40–1.15)	
BMI (kg/m^2^)		0.0000		0.0000
< 18.5	-		-	
18.5– < 25.0	ref		ref	
25.0– < 30.0	5.40 (3.26–8.95)		5.97 (2.90–8.52)	
≥ 30.0	27.04 (17.08–42.81)		25.12 (15.20–41.51)	
Continuous	1.30 (1.26–1.34)		1.23 (1.25–1.34)	
Waist circumference (cm)				
< 102 in men or < 88 in women	ref		ref	
≥ 102 in men or ≥ 88 in women	8.82 (6.28–12.39)		9.23 (6.52–13.07)	
Waist-to-height ratio				
≤ 0.570	ref		ref	
> 0.570	9.44 (6.81–13.09)		8.72 (6.13–12.39)	
Waist-to-hip ratio				
≤ 0.90 in men or ≤0.85 or women	ref		ref	
> 0.90 in men or >0.85 in women	6.65 (4.81–9.21)		6.30 (4.33–9.16)	
Alcohol consumption (daily drinks)		0.0005		0.0006
None	ref		ref	
Light (1 drink/day)	0.59 (0.44–0.78)		0.64 (0.44–0.92)	
Moderate (2 drinks/day)	0.48 (0.34–0.68)		0.47 (0.30–0.73)	
Heavy (> = 3 drinks/day)	0.50 (0.31–0.82)		0.36 (0.19–0.71)	
Physical activity		0.0001		0.0003
Active	ref		ref	
Moderate	2.36 (1.65–3.36)		2.29 (1.47–3.55)	
Inactive	2.60 (1.87–3.60)		2.44 (1.61–3.68)	
Smoking status		0.3438		0.7544
Never	ref		ref	
Former	1.52 (1.12–2.05)		1.02 (0.75–1.39)	
Current	1.11 (0.70–1.75)		1.11 (0.63–1.94)	

*HOMA* homeostasis model assessment, *BMI* body mass index, *CHMS* Canadian Health Measures Survey, *OR* odds ratio

** ORs were adjusted for age, sex, BMI, education, physical activity and alcohol consumption, except for the variable under consideration

** ORs were not adjusted for each other among BMI, waist circumference, waist-to-height ratio and waist-to-hip ratio

Table 3 shows unadjusted and adjusted ORs of IR associated with demographic factors, lifestyle factors and adiposity measures, stratified by gender. The negative association between IR and alcohol consumption was significant only in men but not in women (but the number of heavy drinkers in women was small). The patterns observed for other factors were similar for men and women. Measures of adiposity and physical activity were significantly associated with IR risk, whereas education, income adequacy and smoking were unrelated to IR risk.

**Table 3:** Odds ratios of insulin resistance associated with demographics, adiposity and behavioral factors, by sex, CHMS, Cycle 1 & 2, 2007–2011

Variable	Unadjusted	Adjusted	*p* for trend	Unadjusted	Adjusted	*p* for trend
	OR (95 % CI)	OR (95 % CI) **		OR (95 % CI)	OR (95 % CI) **	
Age (years)			0.0031			0.0194
18–35	ref	ref		ref	ref	
36–50	1.06 (0.60–1.88)	0.85 (0.51–1.42)		1.04 (0.56–1.93)	0.87 (0.41–1.84)	
51–79	2.36 (1.51–3.68)	1.98 (1.28–3.05)		2.00 (1.18–3.38)	1.79 (1.04–3.09)	
Education			0.0959			0.5085
Less than secondary	ref	ref		ref	ref	
Secondary graduate	0.92 (0.46–1.84)	1.09 (0.45–2.62)		0.72 (0.40–1.29)	0.74 (0.40–1.37)	
Other post-secondary	0.44 (0.19–1.01)	0.88 (0.34–2.31)		0.53 (0.20–1.41)	0.77 (0.26–2.29)	
Post-secondary graduate	0.58 (0.28–1.19)	0.60 (0.28–1.29)		0.47 (0.28–0.79)	0.75 (0.44–1.27)	
Family income adequacy			0.0399			0.792
Lowest	ref	ref		ref	ref	
Lower middle	1.74 (0.65–4.65)	1.07 (0.35–3.29)		1.35 (0.67–2.70)	1.10 (0.49–2.46)	
Upper middle	0.80 (0.38–1.70)	0.46 (0.19–1.08)		0.85 (0.57–1.26)	0.73 (0.41–1.32)	
Highest	0.89 (0.44–1.82)	0.50 (0.20–1.25)		0.88 (0.45–1.72)	0.84 (0.40–1.77)	
BMI (kg/m2)			0.0000			0.0000
18.5– < 25.0	ref	ref		ref	ref	
25.0– < 30.0	4.92 (2.18–11.09)	4.47 (2.01–9.94)		5.75 (2.87–11.51)	5.76 (2.62–12.68)	
≥ 30.0	27.69 (12.81–59.85)	25.93 (11.51–58.39)		25.78 (14.63–45.41)	24.85 (13.12–47.06)	
Continuous	1.38 (1.31–1.46)	1.38 (1.30–1.48)		1.25 (1.21–1.30)	1.25 (1.20–1.29)	
Waist circumference (cm)						
< 102 in men or < 88 in women	ref	ref		ref	ref	
≥ 102 in men or ≥ 88 in women	9.97 (6.60–15.04)	8.93 (5.65–14.12)		10.45 (6.91–15.80)	10.42 (6.42–16.92)	
Waist-to-height ratio						
≤ 0.570	ref	ref		ref	ref	
> 0.570	8.83 (5.86–13.29)	7.99 (5.02–12.71)		10.16 (6.74–15.33)	9.82 (6.13–15.73)	
Waist-to-hip ratio						
≤ 0.90 in men or ≤0.85 or women	ref	ref		ref	ref	
> 0.90 in men or >0.85 in women	8.79 (5.83–13.27)	8.21 (4.72–14.28)		5.88 (4.05–8.54)	5.67 (3.60–8.93)	
Alcohol consumption (daily drinks)			0.0038			0.0585
None	ref	ref		ref	ref	
Light (1 drink/day)	0.57 (0.38–0.85)	0.64 (0.38–1.07)		0.55 (0.36–0.83)	0.65 (0.38–1.10)	
Moderate (2 drinks/day)	0.43 (0.28–0.66)	0.42 (0.22–0.83)		0.45 (0.24–0.83)	0.54 (0.23–1.27)	
Heavy (≥3 drinks/day)	0.43 (0.25–0.75)	0.33 (0.15–0.76)		0.27 (0.003–23.2)	0.36 (0.004–34.5)	
Physical activity			0.0572			0.0019
Active	ref	ref		ref	ref	
Moderate	2.32 (1.40–3.85)	2.21 (1.14–4.28)		2.81 (1.21–6.53)	2.65 (1.01–7.00)	
Inactive	2.11 (1.31–3.41)	1.74 (1.05–2.87)		3.94 (2.09–7.42)	3.82 (1.54–9.53)	
Smoking status			0.6536			0.282
Never	ref	ref		ref	ref	
Former	1.47 (1.00–2.17)	0.94 (0.59–1.49)		1.49 (0.95–2.35)	1.07 (0.76–1.51)	
Current	0.78 (0.39–1.55)	0.91 (0.47–1.77)		1.56 (1.01–2.41)	1.38 (0.75–2.54)	

*HOMA* homeostasis model assessment; *BMI* body mass index; *CHMS* Canadian Health Measures Survey; *OR* odds ratio

** ORs were adjusted for age, BMI, education, physical activity and alcohol consumption, except for the variable under consideration

** ORs were not adjusted for each other among BMI, waist circumference, waist-to-height ratio and waist-to-hip ratio

Table 4 presents the unadjusted and adjusted ORs of IR associated with standardized continuous variables, overall and by sex: BMI, WC, WHtR, and WHR. When these four measures were assessed as standardized continuous variables, they were all statistically significantly associated with increased ORs of IR, with their corresponding ORs being 4.20 for BMI, 4.92 for WC, 4.37 for WHtR, and 4.28 for WHR, suggesting that no one measure was superior. However, the ORs for all 4 adiposity measures were slightly higher in men than in women.

**Table 4:** Association of standardized continuous variables with insulin resistance, overall and by sex, CHMS, Cycle 1 & 2, 2007–2011

Variable	Both men and women		Men		Women	
	Unadjusted	Adjusted *	Unadjusted	Adjusted *	Unadjusted	Adjusted *
	OR (95 % CI)	OR (95 % CI)	OR (95 % CI)	OR (95 % CI)	OR (95 % CI)	OR (95 % CI)
Standardized continuous						
BMI (kg/m2)	4.30 (3.57–5.17)	4.20 (3.48–5.08)	6.01 (4.36–8.29)	6.07 (4.23–8.72)	3.52 (2.88–4.29)	3.41 (2.77–4.19)
Waist circumference (cm)	4.73 (3.91–5.72)	4.92 (3.95–6.14)	6.31 (4.38–9.08)	6.18 (4.13–9.23)	4.34 (3.46–5.43)	4.25 (3.40–5.31)
Waist-to-height ratio	4.80 (3.92–5.88)	4.37 (3.90–6.09)	6.52 (4.64–9.17)	6.88 (4.63–10.23)	3.96 (3.19–4.91)	3.97 (3.18–4.96)
Waist-to-hip ratio	2.95 (2.44–3.58)	4.28 (3.08–5.95)	4.36 (3.30–5.74)	5.14 (3.33–7.94)	3.89 (2.91–5.20)	3.98 (2.81–5.66)

*HOMA* homeostasis model assessment, *BMI* body mass index, *CHMS* Canadian Health Measures Survey, *OR* odds ratio

* ORs were adjusted for age, BMI, education, physical activity and alcohol consumption, except for the variable under consideration

* ORs were not adjusted for each other among BMI, waist circumference, waist-to-height ratio and waist-to-hip ratio

## Discussion

This study assessed the association between IR risk and socio-demographics, behavioral factors and several adiposity measures using a sample of non-diabetic adults. Increasing age, being male, being overweight or obese and being physically inactive were all found to be independently associated with a higher risk of IR, whereas education level, family income, and smoking were not significantly associated with IR.

Our study found a significantly increasing risk of IR with age in the non-diabetic adult Canadian population. This finding is comparable to the results in the US [28] and in Spain [29]. Age has been shown to be the most powerful predictor of IR in some studies, but it could be the residual effect of other factors, because diseases or conditions such as obesity, diabetes and hypertension all increase with age. However, a study of Thai adults over 35 years old showed a correlation between IR and age only in women, not in men [30]. The result from the 2246 non-diabetic adults in a representative Spanish population sample suggested a significant nonlinear association with an increase in HOMA-IR index in those women aged 50 years and older, while no evidence existed in men [29]. The molecular mechanisms for the increase of IR with age are not fully understood. There are several aspects of ageing that contribute to increased insulin resistance, including body fat redistribution (decrease in subcutaneous fat and increase in visceral fat), decrease in muscle tissue, increase in pro-inflammatory cytokines, and decreased mitochondrial function [31]. This redistribution of adipose tissue is associated with leptin resistance. This resistance blunts normal central and peripheral functions of leptin, which leads to a decrease in neuroendocrine function and insulin sensitivity, an imbalance in energy regulation, and disturbances in lipid metabolism [32, 33]. Research has showed improved insulin sensitivity by regulating fat metabolism in white and brown adipose tissues by way of caloric restriction or surgical removal of visceral adipose tissue [33–36].

Our study also found a significant difference in IR risk between men and women, which is similar to other reports [29, 37]. This sex difference may be due to differences in adipose tissue distribution, sex hormones and adipokines [37]. Visceral adipocytes have been shown to be more sensitive to catecholamine-induced lipolysis and less sensitive to insulin’s anti-lipolytic effect than are subcutaneous adipocytes [38]. Therefore, increases in visceral and hepatic adipose tissue contribute to dyslipidemia, enhanced gluconeogenesis and insulin insistence [38, 39]. For a given BMI, men have higher lean mass and more visceral and hepatic adipose tissue, whereas women have more general adiposity. In addition, estrogen has been found to have a favorable effect on insulin sensitivity, glucose homeostasis and adipose tissue distribution [37]. Furthermore, compared with women, men have significantly lower level of adiponectin, an insulin-sensitizing hormone [40, 41]. Therefore, greater amounts of visceral and hepatic adipose tissues, in combination with lack of a possible effect of estrogen and lower adiponectin levels, may contribute to men’s higher IR than women.

Our study showed that adiposity indices including BMI, WC, WHR, WHtR were all associated with IR, regardless of gender, which corroborates with other studies [24, 29, 30, 42]. Our study also showed that these four measures of adiposity had similar magnitudes of association. Obesity, especially central obesity, has been demonstrated to be a risk factor for developing insulin resistance [1, 43, 44]. One mechanism is that the excess visceral adipose tissue releases large amount of free fatty acids, which significantly impairs the insulin-signaling pathways in the main target organs [1]. Another mechanism is that inflammatory events decrease the sensitivity to insulin in obese patients [1, 44], with the focuses on adipose tissue macrophages as the main source of obesity-associated inflammation [45]. Inflammatory processes in liver, muscle and other organs also contribute to obesity-induced IR [1].

The negative association between physical activity and IR observed in our study was consistent with other research [24, 29, 42]. It has been demonstrated that physical activity improves substantially insulin sensitivity [46–50]. In addition, physical activity can reduce body fat and obesity by weight loss, which increase cellular insulin sensitivity and reverses IR caused by obesity [46].

Our study also observed a negative correlation between alcohol consumption and IR. This is confirmed in several studies, which reported strong positive associations between alcohol and increased insulin sensitivity [51–53]. Regular low-to-moderate alcohol consumption has been shown to improve insulin sensitivity [51], but chronic heavy alcohol intake may promote insulin resistance [54]. However, there were very few women in the category of heavy drinkers in this study and the negative association between alcohol consumption and IR in women should be interpreted with caution given the wide range of 95 % confidence interval. For this study, because information on separate numbers of drinks of beer, wine and liquor had not been collected, quantity of alcohol intake (grams/day) could not be calculated.

Because IR exists and progresses before pre-diabetes and diabetes could be detected, IR might be the earliest detectable abnormality to predict the development of diabetes and is of clinical relevance. IR could be used a screening tool for early detection of high risk people for diabetes, such as those with high BMI and with abdominal obesity. In addition, IR could be used as a target of therapeutic approach.

### Strengths and limitations

There are several strengths of this study. First, a large national sample of the non-diabetic adult population in Canada was available for this study. This allowed for sufficient power to consider the relationship of a number of variables simultaneously. Second, a number of key variables were measured, not self-reported, thus reducing the possibility of bias. However, there are also some limitations to our study that should be considered when interpreting results. For example, this is a cross-sectional study; therefore, we cannot draw causality from the observed associations among IR and socio-demographics, adiposity and behavioral factors.

In the absence of a universally accepted cut-off point for HOMA-IR, we used an arbitrary cut-off point of the 75 % percentile to define IR, corresponding to a threshold value of 2.61. Previous studies have used the 66th [55], 75th [56, 57], 80th [58] and 90th [30, 59, 60] percentile. Three studies used receiver operator characteristic (ROC) curves to establish their cut-off points [61–63]; while this is preferable, it requires information on sensitivity and specificity which can only be obtained when data from insulin clamp testing is also available. HOMA-IR threshold values from these studies ranged from 1.55 in a south-east Asian population [30] to 3 in a Spanish population aged 7–16 years [62]. A large multinational study involving 17 European and 2 American sites noted a 23 % prevalence of insulin resistance based on insulin clamp, similar to our classification of the top 25 % of our population as insulin resistant [64]. A major limitation of this study is that the cut-off point used has not been validated with the gold standard for the Canadian population.

Furthermore, because this study combined data from two consecutive cycles, study methods and assay procedures may have introduced small non-differential variation across the two cycles. In addition, the two cycles of the CHMS have only a modest response rate, which could affect the representation of the Canadian population, although this level of response rate is common in other surveys in current time.

## Conclusion

In summary, the current study demonstrated a positive association for obesity and a negative association for physical activity with IR. The current study results, particularly the elevated risk of IR observed in obese people suggests that early interventions such as weight loss and physical activity may be important in preventing diabetes. With the high prevalence of overweight and obesity in the Canadian population, the study of IR could be considered an important research and public health topic.

## Abbreviations

BMI, body mass index; CHMS, the Canadian Health Measures Survey; HOMA, homeostasis model assessment of insulin resistance; IR, insulin resistance; WC, waist circumference; WHR, waist-to-hip ratio; WHtR, waist-to-height ratio
